# Efficacy of ZnO nanoparticles in Zn fortification and partitioning of wheat and rice grains under salt stress

**DOI:** 10.1038/s41598-022-26039-8

**Published:** 2023-02-04

**Authors:** Zuhra Mazhar, Javaid Akhtar, Aiyeshah Alhodaib, Tayyaba Naz, Mazhar Iqbal Zafar, Muhammad Mazhar Iqbal, Humaria Fatima, Iffat Naz

**Affiliations:** 1 Institute of Soil and Environmental Sciences, University of Agriculture, Faisalabad, 38040 Pakistan; 2 Saline Agriculture Research Centre, University of Agriculture, Faisalabad, 38040 Pakistan; 3 Department of Physics, College of Science, Qassim University, Buraydah, 51452 Saudi Arabia; 4 Department of Environmental Sciences, Faculty of Biological Sciences, Quaid-i-Azam University, Islamabad, 45320 Pakistan; 5 Soil and Water Testing Laboratory, Department of Agriculture, Ayub Agricultural Research Institute, Government of Punjab, Chiniot, 35400 Pakistan; 6 Department of Pharmacy, Faculty of Biological Sciences, Quaid-i-Azam University, Islamabad, 45320 Pakistan; 7 Department of Biology, Science Unit, Deanship of Educational Services, Qassim University, Buraydah, 51425 Saudi Arabia

**Keywords:** Plant sciences, Environmental sciences, Materials science, Nanoscience and technology

## Abstract

Zinc (Zn) deficiency is a major health concern in developing countries due to dependency on cereal based diet. Cereals are inherently low in Zn and inevitable use of stressed land has further elevated the problem. The aim of current research was to improve wheat and rice grains grain Zn concentration grown in saline soils through zinc oxide nanoparticles (ZnO-NPs) due to their perspective high availability. The ZnO-NPs were prepared by co-precipitation method and characterized through X-ray diffraction (XRD) and Scanning Electron Microscope (SEM). Two separate pot experiments for wheat and rice were conducted to check the relative effectiveness of ZnO-NPs compared to other bulk Zn sources i.e., zinc sulphate heptahydrate (ZnSO_4_·7H_2_O) and ZnO. Results showed that salt stress negatively impacted the tested parameters. There was a significant (p ≤ 0.05) improvement in growth, salt tolerance, plant Zn uptake and grain Zn concentrations by Zn application through Zn sources. The ZnO-NPs showed maximum improvement in crops parameters as compared to other sources due to their higher uptake and translocation in plants under both normal and stressed soil conditions. Thus, ZnO nanoparticles proved to be more effective for grain Zn fortification in both tested wheat and rice crops under normal and saline conditions.

## Introduction

Salinity has been a major concern to global agriculture and has become the most threatening abiotic environmental stress resulting in loss of fertility and crop productivity^1^. Increased concentrations of sodium (Na^+^) and chloride (Cl^−^) and osmotic stress leads to reduced absorption of essential nutrients, reduced water availability and functional disorders of several physiological processes of plants^2,3^. Hence crop yield and quality is greatly compromised. Ionic imbalance and high concentration of Na^+^ in plants results in retarded growth and poor nutrient content of cereals^4^ including wheat and rice. Occurrence of few or all of the factors like arid climate, high regional temperature resulting in net uphill water movement and surface salt accumulation, imbalanced and insufficient fertilizer usage, low organic matter (OM), high pH, calcareous soil and high carbonate irrigation water etc. leads to soil salinity and deficiency of most nutrients including Zn^5^.

Zinc has been assessed as the most commonly deficient micronutrient in most calcareous soils after N and P^6^. Commonly grown cereal crops, wheat and rice are most likely to suffer from Zn deficiency in developing countries^7^. The Zn is a vital component of several enzymes and acts as a cofactor of more than 300 enzymes. In plants Zn is needed for translocation, transcription and regulation of most enzymatic activities and is vital for structural stability of several proteins^8^ and structural component of ribosomes^7^.

About half of the Asian and African countries population is at risk of Zn deficiency and the rate is increasing at an alarming scale^9^. The Zn deficiency is more common in women and children due to severe malnutrition. Dependence on the cereal-based diet with very low Zn concentration and low bioavailability is the main reason behind this scenario. Increasingly deteriorating soils further worsen the issue and it cannot meet the human need for sufficient Zn uptake. Saline and/or sodic soils have reduced solubility of micronutrients, so plants grown on such soils have to face the deficiency of micronutrients especially Zn^10^. It is reported that Zn has an important role in stress alleviation and helps in reduced plant Na^+^ and higher plant K^+^ accumulation under saline conditions^11^.

Zinc deficiency and salt stress are usually discussed as two separate growth limiting factors while, their interaction effect is not studied in details and not well reported. However, few researchers have documented the effect of salt stress on Zn uptake. Soils with high SAR and pH have very low solubility of micronutrients^12^. High ionic strength of growth medium has a high negative affect on plant Zn uptake.

Wheat and rice both are most important staple food especially in developing countries of South-East Asia and people of these countries rely on these two cereals for major part of their daily calorie intake. Both of these cereals are considered as poor source of Zn in terms of bioavailability and total Zn contents. Most commonly used cereals like wheat and rice were reported to suffer from Zn deficiency in calcareous soils^5,6^. The situation becomes worse when there is a problem of salt stress.

In view of current scenario, we need to maintain adequately large soil pool of plant available Zn. For that we strongly need development and application of new fertilizer technologies to provide nutritious crops to this rapidly increasing global population. The present study is focused on the agronomic fortification of wheat and rice with Zn. Agronomic fortification has been proved very effective for cereals especially wheat and rice. Given the fact that a higher concentration of Zn is required to achieve a computable impact on human health and also to avoid any yield loss in plants due to deficiency of Zn. Hence, for crop Zn biofortification, providing sufficient Zn through fertilizers by different means is critically important.

In these regards, use of nanotechnology can be an effective way to deal with the situation. Due to the smaller size and higher surface area^13^, NPs have many potential applications in agriculture including nano-fertilizers^14^. Among engineered nano-materials, zinc oxide nanoparticles (ZnO-NPs) are a commonly used metal oxide nano particles. Nano-ZnO is also one of the Zn compounds listed as “generally recognised as safe” (GRAS) by USFDA (United States Food and Drug Administration)^15^. The ZnO nanoparticles normally appear as white powder. It is sparingly soluble in water. Due to their smaller size and large surface area, ZnO nanoparticles are expected to be the ideal replacement for conventional Zn fertilizers for plants^16^.

Keeping in view these current scenarios and issues, present study was set up to understand reactions of ZnO nanoparticles in soil plant system so that an evaluation can be made for its possible use as a more efficient fertilization option as compared to available bulk resources of Zn. Mainly evaluating the effectiveness of ZnO-NPs in salt stressed conditions due to perspective unavoidable use of stressed lands for cereals growth.

## Materials and methods

### Synthesis of ZnO nanoparticles

**Figure 1:**
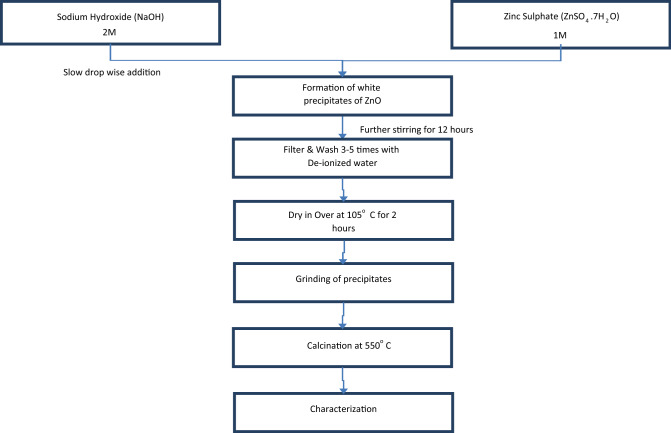
Flow chart of ZnO nanoparticles synthesis.

The ZnO nanoparticles were prepared by co-precipitation method. A proposed procedure was followed with slight modifications^17^. Briefly, freshly prepared NaOH solution was slowly added to the solution of ZnSO_4_·7H_2_O in drop wise manner at 2:1 ratio respectively. Resulting milky white mixture was stirred for 12 h on magnetic stirrer. Prepared ZnO precipitates were filtered (Whatman No. 42) and then washed thoroughly with deionized water. Washing and filtration was done at least thrice to completely wash the precipitates. Afterwards, precipitates were dried at 105 °C in a forced air oven. Dried precipitates were ground in a pestle and mortar and calcined at 550 °C for 2 h. Step wise method is presented in Fig. 1. Balanced reaction equation is as follows:

$$2{\text{NaOH }} + {\text{ ZnSO}}_{4} \cdot 7{\text{H}}_{2} {\text{O}} \to {\text{ZnO }}\left( {{\text{ppt}}} \right) \, + {\text{ Na}}_{2} {\text{SO}}_{4} + \, 8{\text{H}}_{2} {\text{O}}.$$

X-ray powder diffraction (XRD), Zeta sizer and Scanning electron microscopy (SEM) analysis were used to characterize the prepared nanoparticles^18^.

### Characterization of synthesized nanoparticles

#### X-ray powder diffraction (XRD)

X-ray diffractometer analysis was done to determine the crystalline- phase structure and the size of ZnO-NPs. The ZnO NPs crystal size was calculated by the Debye–Scherrer equation^19^:

$$D=\frac{k\lambda }{\beta \mathrm{cos}\theta },$$

where, D = the mean crystalline size, *k* = Scherer constant (0.89), λ = X-ray wavelength, β = full width of half peak maximum (FWHM) intensity (in radians) denoted as $\Delta \left(2\uptheta \right)$ and θ = Bragg’s diffraction angle.

### ZnO nanoparticles suspension preparation

For each application of nanoparticles, already weighed amount of required ZnO nanoparticles for wheat and rice (Table 2) was suspended directly in deionised water in a flask and then particles were dispersed through ultrasonic vibration in a water bath sonicator for 30 min just before the application of treatment. Each replication and treatment was sonicated separately.

### Pot experiments

Wheat cultivar FSD-2008 was obtained from Wheat Research Institute Faisalabad and Rice cultivar IR-6 was used and got from Rice Research Institute Kala Shah Kaku. Both are approved varieties and permission was granted to use them for experimental purposes from respective research stations. Two separate pot experiments for wheat and rice crops were arranged in wire house of Institute of Soil and Environmental Sciences, University of Agriculture Faisalabad.

### Growth conditions and crops husbandry practices

Normal surface soil (0–20 cm) was collected from agricultural fields of land Utilization Farm, University of Agriculture Faisalabad (UAF) Pakistan. Pre sowing analyses of soil were done following standard methods (Table 1). Pots filling were done at rate of 12 kg soil per pot for wheat and 8 kg per pot for rice. Salinity was developed by mixing calculated amount of NaCl in soil of each pot prior to pot filling. The Zn was applied using three sources (ZnSO_4_·7H_2_O, ZnO and ZnO nanoparticles) for each crop. Treatment plan for wheat and rice experiment is described in Table 2.

**Table 1:** Pre-sowing analysis of the soil used for present study.

Soil characteristic	Unit	Value
pH_s_	–	7.71
EC_e_	dS m^−1^	2.28
OM	%	0.40
Saturation percentage	%	31.1
Textural class	–	Sandy clay loam
Sand	%	49.5
Silt	%	26.6
Clay	%	23.9
Total N	%	0.041
Available P	mg kg^−1^	7.7
Extractable K	mg kg^−1^	110.2
DTPA extractable Zn	mg kg^−1^	1.10
DTPA extractable Fe	mg kg^−1^	5.09
CO_3_^2−^	(mmolc L^−1^)	0.69
HCO_3_^−^	(mmolc L^−1^)	7.98
Cl^−^	(mmolc L^−1^)	4.45

**Table 2:** Treatment plan.

Symbol	Treatment for wheat experiment	Treatment for rice experiment
T_1_	Control	Control
T_2_	7 mg Zn kg^−1^ as ZnSO_4_·7H_2_O (369.46 mg ZnSO_4_·7H_2_O per12 kg pot)	10 mg Zn kg^−1^ as ZnSO_4_·7H_2_O (353.92 mg ZnSO_4_·7H_2_O per 8 kg pot)
T_3_	7 mg Zn kg^−1^ as ZnO (105.12 mg ZnO per 12 kg pot)	10 mg Zn kg^−1^ as ZnO (100.16 mg ZnO per 8 kg pot)
T_4_	7 mg Zn kg^−1^ as ZnO-NPs (105.12 mg ZnO-NPs per 12 kg pot)	10 mg Zn kg^−1^ as ZnO-NPs (100.16 mg ZnO-NPs per 8 kg pot)
T_5_	10 dS m^−1^ EC	7 dS m^−1^ EC
T_6_	10 dS m^−1^ EC + 7 mg Zn kg^−1^ as ZnSO_4_·7H_2_O (369.46 mg ZnSO_4_·7H_2_O per 12 kg pot)	7 dS m^−1^ EC + 10 mg Zn kg^−1^ as ZnSO_4_·7H_2_O (353.92 mg ZnSO_4_·7H_2_O per 8 kg pot)
T_7_	10 dS m^−1^ EC + 7 mg Zn kg^−1^ as ZnO (105.12 mg ZnO per 12 kg pot)	7 dS m^−1^ EC + 10 mg Zn kg^−1^ as ZnO (100.16 mg ZnO per 8 kg pot)
T_8_	10 dS m^−1^ EC + 7 mg Zn kg^−1^ as ZnO-NPs (105.12 mg ZnO-NPs per 12 kg pot)	7 dS m^−1^ EC + 10 mg Zn kg^−1^ as ZnO-NPs (100.16 mg ZnO-NPs per 8 kg pot)

Seeds were sown directly in case of wheat, while nursery was raised for rice in field and then 30 days old seedlings were transplanted in treated pots. Pots were arranged in completely randomized design and each treatment was replicated thrice. Urea (46% N), di-ammonium phosphate (DAP, 46% P_2_O_5_, 18% N) and potassium sulphate (SOP, 50% K_2_O) were used as fertilizer sources of NPK respectively. Complete dose of P and K was applied at sowing (transplanting in case of rice) while, half of the N was applied at sowing/transplanting and other half was applied at early flowering stage. For wheat, 0.52 g urea, 2.68 g DAP and 0.86 g SOP were used for each 12 kg pot while, for rice, 0.313 g urea, 1.20 g DAP and 0.58 g SOP were used for each 8 kg pot.

Total chlorophyll content (TCC) index value in terms of Special Products Analysis Division (SPAD, a division of Minolta) were determined at flag leaf stage using a hand-held SPAD-502m (Minolta, Osaka, Japan). Young fully expanded leaves were selected from apex to get readings. Three readings were taken from leaf tip to leaf base and the average was taken^20^.

Crops were harvested at maturity and growth parameters like plant height, straw yield, grain yield, No. of tillers, No. of spikes and spikelet were recorded. Grain and straw samples were collected and dried in a fan forced oven at 65 ± 5 °C for 72 h or unless the constant weight is achieved for further chemical analysis.

### Ionic parameter measurements

Dried plant samples were ground in a mechanical grinder to powder form and stored in zip locked plastic bags. Finely ground and dried plant samples were digested according to modified wet digestion procedure^21^ for high recovery of Zn.

### Na^+^, K^+^ and Zn determination

The Na^+^ and K^+^ were determined via flame photometer (Jenway PFP-7, Loughborough, Leicestershire, UK), whereas Zn was determined via flame atomic absorption spectrophotometer (FAAS; Model Thermo S-Series, Thermo Electron Corporation, Cambridge, UK) following the procedures mentioned in ICARDA (International Centre for Agricultural Research in the Dry Areas) manual by Estefan et al.^22^. For Zn determination of rice, husk from the paddy was removed to get results for rice grain.

### Zn uptake and percentage

Zn uptake of root, shoot and grain was calculated by using following formula:

$$\mathrm{Zn }uptake \left(mg\, {plant}^{-1}\right)=\frac{[\left(\mathrm{Zn }concentration \,in \,plant \,part\left(\mathrm{mg g}^{-1}\right) \times dry \,weight \,of \,plant\, part\,\left(\mathrm{g}\right)\right]}{Number \,of\, plants\, per\, pot}.$$

Zn percentage in each plant part was calculated by following formula:

$$Zn\,{ \%}age\, uptake\, in\, plant part=\frac{\mathrm{ZnX}}{\mathrm{ZnY}}\times 100,$$

where ZnX is Zn uptake in specific plant part (root, shoot and grain) mg plant^−1^ and ZnY is sum of Zn uptake in plant root, shoot and grain (mg plant^−1^).

## Results

### Characterization of ZnO nanoparticles

**Figure 2:**
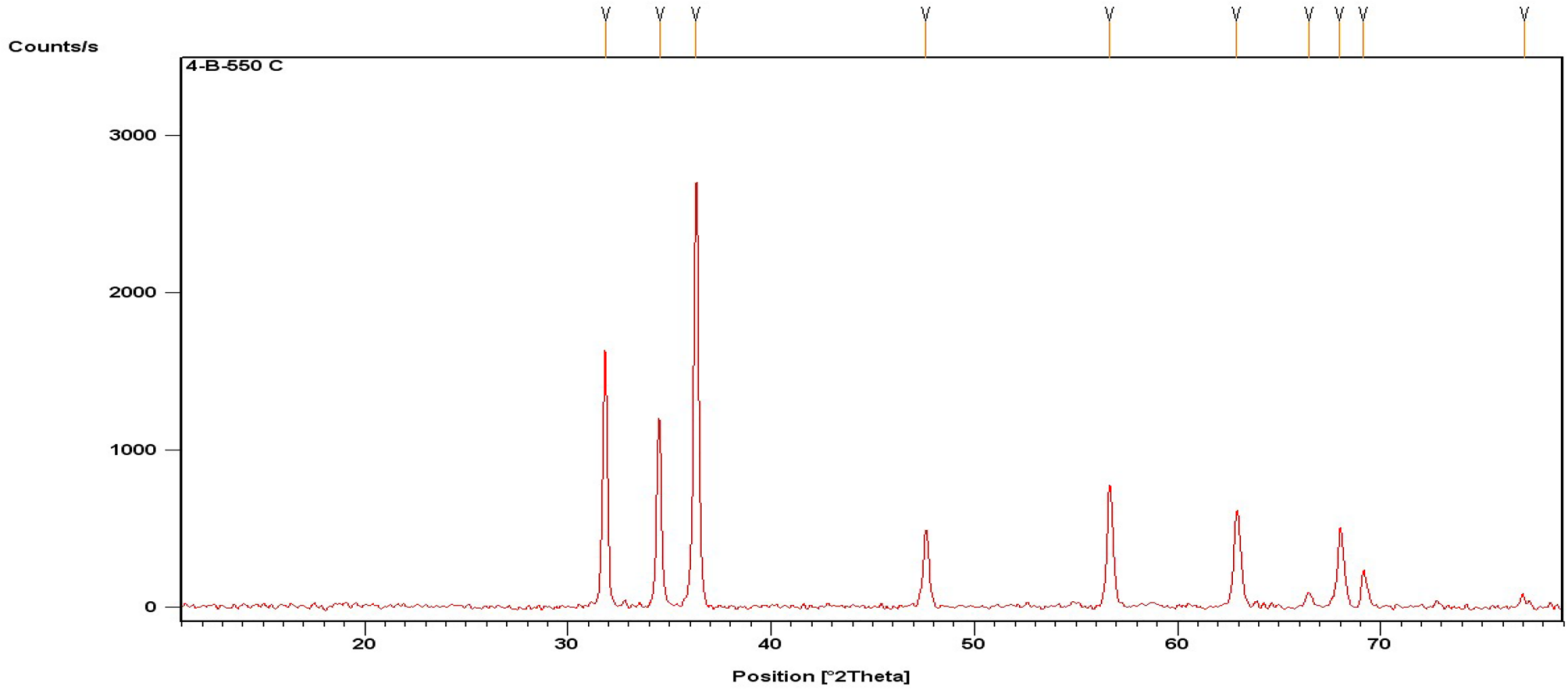
X-ray diffraction pattern of ZnO-NPs.

**Figure 3:**
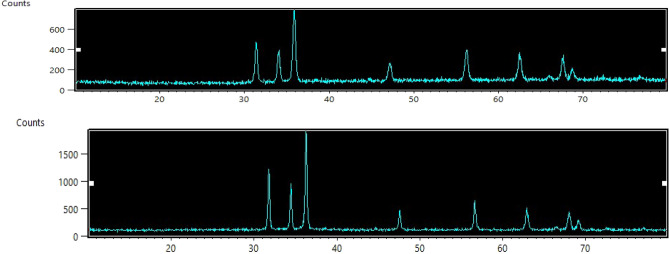
X-ray diffraction pattern of ZnO-NPs.

The ZnO-NPs with an average particle size of about 20–60 nm were calculated using Scherrer equation. Characterization of synthesized particles was done by XRD and SEM. The XRD analysis was done to determine the purity and crystalline size of synthesized ZnO-NPs. Pattern of ZnO-NPs X-ray diffraction is represented in Figs. 2 and 3. All the peaks represented in diffraction pattern matched well with the crystal planes of the hexagonal wurtzite ZnO structure (JCPDS card No. 36-1451) as the location of diffraction peaks can be seen at diffraction angles (2°Th.) 31.8°, 34.5°, 36.3°, 47.6°, 56.6°, 62.9°, 66.4°, 67.9°, 69.1° and 77° that correspond well to it^23,24^.

All the diffraction peaks showed sharp peak intensities that indicate that prepared material has good crystalline nature and consists of particles in nano range. It also confirmed the purity of synthesized ZnO-NPs as there were no traces of peaks recorded other than ZnO ones. The prepared ZnO-NPs diameter was calculated by Scherrer equation^19^ was found to be 22.96 nm where *β* is the FWHM of diffraction peak corresponding to plane (101) located at 36.3°. The SEM images at 20 kX also showed a size of 51 nm particles with spherical shape.

### Growth and yield responses of wheat

Data for growth and yield parameters e.g. Plant height (PH), No. of tillers (T), grain yield (GY) and total chlorophyll contents (TCC) of wheat are listed in Table 3.

**Table 3:** Comparative effect of ZnO-NPs to other bulk Zn sources on growth and yield attributes of wheat.

Treatment	Plant height (cm)	Tillers (per pot)	Grain yield (g pot^−1^)	Total chlorophyll contents (SPAD-value)
T_1_ = Control	82.8 ± 0.73	15.3 ± 0.33	25.7 ± 0.26	50.7 ± 0.041
T_2_ = ZnSO_4_·7H_2_O	86 ± 1.44 (3.82)	17.7 ± 0.33 (15.22)	31.4 ± 0.32 (22.24)	53.9 ± 1.36 (6.26)
T_3_ = ZnO	83.5 ± 0.58 (0.8)	16.7 ± 0.33 (8.7)	29.3 ± 0.18 (14.07)	51.2 ± 0.52 (1.07)
T_4_ = ZnO-NPs	87.8 ± 0.44 (6.04)	18 ± 0.58 (17.39)	33.8 ± 0.45 (31.53)	54.4 ± 0.47 (7.25)
T_5_ = 10 dS m^−1^ EC	75.3 ± 1.48	7.3 ± 0.33	11.5 ± 0.57	39.6 ± 0.47
T_6_ = 10 dS m^−1^ EC + ZnSO_4_·7H_2_O	77.8 ± 0.83 (3.32)	8.7 ± 0.33 (18.18)	16.3 ± 0.48 (41.73)	47.5 ± 1.12 (19.99)
T_7_ = 10 dS m^−1^ EC + ZnO	77.3 ± 0.67 (2.65)	8.3 ± 0.33 (13.64)	14.8 ± 0.33 (28.59)	43.5 ± 0.77 (9.72)
T_8_ = 10 dS m^−1^ EC + ZnO-NPs	78.2 ± 0.6 (3.76)	9.7 ± 0.67 (31.82)	18.5 ± 0.32 (60.5)	47.6 ± 0.84 (20.02)
**F-values**				
Salinity	249.1***	978.2***	2256.86***	169.68***
Zn source	11.8***	15.7***	116.34***	20.69***
Salinity × Zn source	2.1^ns^	0.6^ns^	1.58^ns^	3.03^ns^

Values represent mean ± SE (n = 3). Values in parenthesis show percent increase or decrease from respective control. According to LSD test with multiple comparisons, F-values of the Two-way ANOVA are indicated as:ns not significant.

*Significant at p ≤ 0.05.

**Significant at p ≤ 0.01.

***Significant at p ≤ 0.00.

Analysis of variance for data of all these parameters showed a significant difference (p ≤ 0.05) among sources and salt treatments, while interaction effects were not significant for all these parameters. That means although there is a difference in behaviour of all three sources in both growth conditions (normal and saline), the respective behaviour of every source was almost the same with respect to growth conditions (either normal or saline). Plant growth was greatly affected under salt stress but significantly improved through the application of Zn. The response of plants to the Zn application through each source was different. The maximum increase in growth concerning respective control was showed by ZnO-NPs followed by ZnSO_4_·7H_2_O and least percentage increase was given by ZnO application in bulk form in both saline and normal growth conditions.

### Na^+^ and K^+^ contents of wheat

Data presented in Table 4 regarding shoot Na^+^ and K^+^ concentration showed that Na^+^ concentration was highly increased under saline conditions for all applied treatments, whereas K^+^ concentration was decreased. Zinc application through all sources significantly improved the situation. Maximum decrease in shoot Na^+^ (30%) was showed by ZnSO_4_·7H_2_O under normal conditions. While, under saline conditions maximum decrease (27%) in shoot Na^+^ was recorded by ZnO-NPs. A significant effect (p ≤ 0.05) of different Zn source and salt treatment on Na^+^ and K^+^ concentration was evident in variance analysis.

**Table 4:** Comparative effect of ZnO-NPs to other bulk Zn sources on salt tolerance attributes (g kg^−1^ dry wt) and Zn concentration (mg kg^−1^ dry wt) of wheat.

Treatment	Shoot Na^+^ (g kg^−1^ dry wt)	Shoot K^+^ (g kg^−1^ dry wt)	Shoot Zn (mg kg^−1^ dry wt)	Root Zn (mg kg^−1^ dry wt)	Grain Zn (mg kg^−1^ dry wt)
T_1_ = Control	0.60 ± 0.01	11.86 ± 0.22	25.9 ± 0.94	33.9 ± 2.7	25.6 ± 2.4
T_2_ = ZnSO_4_·7H_2_O	0.41 ± 0.003 (− 30.8)	14.9 ± 0.24 (25.66)	44.3 ± 1.13 (71.22)	49.6 ± 2.71 (46.27)	46.6 ± 1.6 (82.04)
T_3_ = ZnO	0.53 ± 0.005 (− 10.85)	14.3 ± 0.07 (20.86)	39 ± 1.07 (50.61)	46.2 ± 3.1 (36.36)	39.3 ± 0.19 (53.54)
T_4_ = ZnO-NPs	0.45 ± 0.023 (− 25.79)	15.2 ± 0.37 (28.34)	51 ± 0.74 (97.11)	54.1 ± 1.6 (59.57)	52.9 ± 1.5 (106.43)
T_5_ = 10 dS m^−1^ EC	1.42 ± 0.073	7.25 ± 0.05	15.7 ± 2.03	27.4 ± 2.6	18.5 ± 1.3
T_6_ = 10 dS m^−1^ EC + ZnSO_4_·7H_2_O	1.14 ± 0.036 (− 20.07)	8.65 ± 0.26 (19.38)	29.5 ± 0.71 (87.35)	41.4 ± 0.32 (51.14)	37 ± 0.59 (99.69)
T_7_ = 10 dS m^−1^ EC + ZnO	1.25 ± 0.009 (− 12.41)	7.7 ± 0.18 (6.22)	23.4 ± 0.85 (48.58)	36.3 ± 1.36 (32.28)	26.4 ± 0.8 (42.5)
T_8_ = 10 dS m^−1^ EC + ZnO-NPs	1.04 ± 0.029 (− 27.01)	8.85 ± 0.20 (22.18)	34.9 ± 1.05 (121.68)	48.3 ± 1.28 (76.14)	45.7 ± 1.45 (146.45)
**F-values**					
Salinity	917.72***	2184***	280.06***	48.67***	92.29***
Zn source	26.9***	76.33***	123.95***	63.71***	149.27***
Salinity × Zn source	4.11*	12.91**	2.61^ns^	0.73^ns^	2.03^ns^

Values represent mean ± SE (n = 3). Values in parenthesis show percent increase or decrease from respective control. According to LSD test with multiple comparisons, F-values of the Two-way ANOVA are indicated as: *ns* not significant.

*Significant at p ≤ 0.05.

**Significant at p ≤ 0.01.

***Significant at p ≤ 0.001.

Interaction effect of salt imposition and Zn application were also significant in all these parameters. Similarly, maximum increase in shoot K^+^ (28 and 22%) was recorded under ZnO-NPs treatment under normal and saline conditions respectively. Minimum increase in shoot K^+^ was recorded where ZnO in bulk form was applied. Normally there was a substantial difference (p ≤ 0.05) between response of ZnO bulk and other two sources. While, difference in responses of ZnSO_4_·7H_2_O and ZnO-NPs was less evident in few parameters. K^+^/Na^+^ ratio was also increased in a positive manner due to Zn application in both normal and saline conditions.

### Plant Zn^2+^ concentration and translocation in wheat

**Figure 4:**
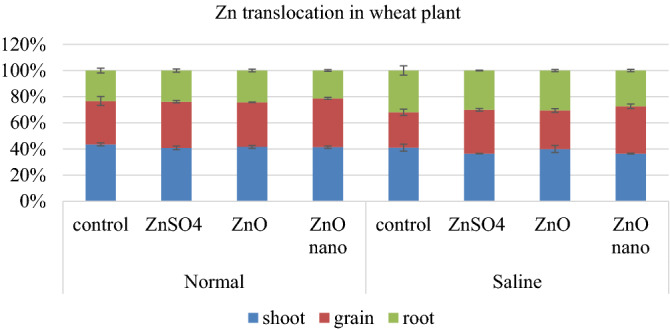
Percentage distribution of Zn translocated in different plant parts of wheat at maturity (Each value is a mean, n = 3 statistically significant at p ≤ 0.05, T bars represent ± standard error of means).

Analysis of Variance for Zn concentration showed that response of wheat to Zn application was highly significant (p ≤ 0.05) and a substantial difference existed among Zn uptake through different sources of applied Zn. In normal soil, the Zn concentration in grain was 25.6 mg kg^−1^ without Zn application (Table 4). The maximum Zn concentration in grain (52.9 mg kg^−1^) was observed in treatment where ZnO-NPs were applied. Under saline conditions, without Zn application, the Zn concentration in grains was 18.5 mg kg^−1^, which was increased to 45.7 mg kg^−1^ with the application of ZnO-NPs. Similar trend was observed in shoot and root Zn concentrations. The interaction of salinity × Zn source was highly significant for Zn uptake per plant in all three components of wheat (shoot, root and grain). As for Zn partitioning in each plant part more Zn was translocated to grain where ZnO-NPs were applied under both normal and saline conditions. The Zn translocation in each plant part of wheat is depicted in Fig. 4, which represents the percentage of total Zn translocated in each plant part.

### Growth, yield and physiological responses of rice

Data for responses of different growth parameters like, shoot length, No. of tillers, paddy yield and total chlorophyll contents (TCC) of rice is presented in Table 5. Results for growth responses are almost in accordance to the responses we got for wheat plants. Responses of all the parameters were significantly positive towards Zn application in both normal and saline conditions. But the interaction effects were only significant for paddy yield.

**Table 5:** Comparative effect of ZnO-NPs to other bulk Zn sources on growth attributes of rice.

Treatment	Shoot length (cm)	Tillers (per pot)	Paddy yield (g pot^−1^)	Total chlorophyll contents (SPAD-value)
T_1_ = Control	84.3 ± 1.4	13 ± 0.43	15.5 ± 0.53	47.9 ± 1.42
T_2_ = ZnSO_4_·7H_2_O	91.3 ± 1.5 (8.32)	17.7 ± 0.75 (35.29)	23.4 ± 1.04 (51.29)	51.6 ± 0.68 (7.79)
T_3_ = ZnO	87.7 ± 2.8 (3.96)	14 ± 0.66 (7.84)	21 ± 0.79 (35.78)	49 ± 1.95 (2.3)
T_4_ = ZnO-NPs	94.3 ± 2.2 (12.08)	17.7 ± 0.50 (37.25)	25.5 ± 0.58 (64.66)	50.3 ± 1.33 (5.08)
T_5_ = 7 dS m^−1^ EC	68 ± 1.01	7.8 ± 0.25	6.4 ± 0.72	34.4 ± 0.95
T_6_ = 7 dS m^−1^ EC + ZnSO_4_·7H_2_O	73.7 ± 1.04 (8.35)	10.8 ± 1.52 (38.71)	10.5 ± 0.42 (63.87)	41 ± 0.58 (19.19)
T_7_ = 7 dS m^−1^ EC + ZnO	72.3 ± 2.5 (6.39)	9.3 ± 0.33 (20.43)	9.3 ± 0.33 (45.03)	39 ± 0.58 (13.37)
T_8_ = 7 dS m^−1^ EC + ZnO-NPs	74.3 ± 2.2 (9.09)	9.7 ± 0.67 (24.73)	11.9 ± 0.45 (84.89)	40.9 ± 1.21 (18.8)
**F-values**				
Salinity	148.53***	114.78***	702.23***	160.41***
Zn source	6.17**	9.73**	55.21***	7.1**
Salinity × Zn source	0.5^ns^	1.86^ns^	5.06*	1.09^ns^

Values represent mean ± SE (n = 3). Values in parenthesis show percent increase or decrease from respective control. According to LSD test with multiple comparisons, F-values of the Two-way ANOVA are indicated as: *ns* not significant.

*Significant at p ≤ 0.05.

**Significant at p ≤ 0.01.

***Significant at p ≤ 0.001.

In general growth, maximum tillers were produced by ZnSO_4_·7H_2_O under saline conditions while all other parameters showed the maximum increase under ZnO-NPs (T_8_) followed by the application of ZnSO_4_·7H_2_O. While, the minimum percentage increase was observed under application of ZnO bulk application. In normal soil conditions the difference between T_2_ and T_4_ is generally non-significant.

In case of yield responses maximum paddy yield was recorded in T_4_ (26 g pot^−1^) followed by T_2_ (23 g pot^−1^) in normal conditions and T_8_ (12 g pot^−1^) followed by T_6_ (10 g pot^−1^) in saline conditions. Chlorophyll contents in terms of SPAD value were significantly increased when Zn treatments were applied in both normal and saline conditions. But the difference among the sources was not significant.

### Na^+^ and K^+^ concentration of rice

In present pot culture study plant Na^+^ concentration, K^+^ concentration, and K^+^/Na^+^ ratio (Table 6) were significantly (p ≤ 0.05) affected by salt imposition. Na^+^ concentration was highly increased under salt stress while K^+^ concentration and K^+^/Na^+^ ratio was decreased substantially under saline conditions. Zn application improved the negative aspects of salt stress by increasing K^+^ concentration and vice versa for Na^+^ concentration in all plant parts. Variance analysis of data showed that there exists a significant difference (p ≤ 0.05) among sources towards shoot and root K^+^ and Na^+^ concentration in both normal and saline soil conditions. But interaction effects (salinity × source) for both shoot K^+^ and Na^+^ concentrations were not significant. The ZnO-NPs gives better results under both normal and saline soil conditions as compared to other sources (ZnSO_4_·7H_2_O and ZnO).

**Table 6:** Comparative effects of ZnO-NPs to other bulk Zn sources on salt tolerance attributes (g kg^−1^ dry wt) and Zn concentration (mg kg^−1^ dry wt) of rice.

Treatment	Shoot Na^+^ (g kg^−1^ dry wt)	Shoot K^+^ (g kg^−1^ dry wt)	Shoot Zn (mg kg^−1^ dry wt)	Root Zn (mg kg^−1^ dry wt)	Grain Zn (mg kg^−1^ dry wt)
T_1_ = Control	1.26 ± 0.058	10.2 ± 0.14	22.2 ± 1.12	32.6 ± 0.91	30.4 ± 1.5
T_2_ = ZnSO_4_·7H_2_O	0.89 ± 0.041 (− 29.69)	11.8 ± 0.18 (16.61)	32.6 ± 0.67 (46.7)	44.5 ± 1.53 (36.47)	42.7 ± 1.8 (40.2)
T_3_ = ZnO	1.05 ± 0.019 (− 16.96)	10.6 ± 0.16 (4.4)	28.3 ± 0.85 (27.06)	38 ± 0.68 (16.65)	37.4 ± 1.1 (22.9)
T_4_ = ZnO-NPs	0.72 ± 0.11 (− 43.04)	12.2 ± 0.27 (20.29)	34.3 ± 0.31 (54.06)	45.5 ± 0.95 (39.68)	45 ± 1.3 (47.73)
T_5_ = 7 dS m^−1^ EC	2.93 ± 0.08	3.9 ± 0.36	15.5 ± 0.57	19.2 ± 0.20	23.3 ± 0.67
T_6_ = 7 dS m^−1^ EC + ZnSO_4_·7H_2_O	2.39 ± 0.014 (− 18.31)	5.7 ± 0.12 (46)	22.8 ± 0.81 (47.45)	26.1 ± 0.84 (36.02)	30.8 ± 1.3 (31.98)
T_7_ = 7 dS m^−1^ EC + ZnO	2.61 ± 0.07 (− 11.02)	4.5 ± 0.31 (16.83)	19.5 ± 0.36 (25.82)	22.1 ± 0.43 (15.1)	26.7 ± 1.28 (14.52)
T_8_ = 7 dS m^−1^ EC + ZnO-NPs	2.23 ± 0.09 (− 23.77)	5.8 ± 0.25 (49.69)	25.5 ± 0.92 (64.96)	29.2 ± 0.67 (52.38)	31.9 ± 1.05 (36.74)
**F-values**					
Salinity	1049***	1440***	229.93***	637.18***	153.6***
Zn source	30.58***	33.96***	73.79***	66.86***	36.27***
Salinity × Zn source	0.59^ns^	0.19^ns^	1.28^ns^	2.59^ns^	2.23^ns^

Values represent mean ± SE (n = 3). Values in parenthesis show percent increase or decrease from respective control. According to LSD test with multiple comparisons, F-values of the Two-way ANOVA are indicated as: *ns* not significant.

*Significant at p ≤ 0.05.

**Significant at p ≤ 0.01.

### Plant Zn concentration and translocation in rice

There was a significant difference (p ≤ 0.05) among different Zn sources in plant Zn concentration. A profound increase in the shoot, grain, and root Zn concentration (Table 6) of rice plant with Zn application under both (normal and saline) growth conditions was recorded. Shoot Zn was increased from 22 mg kg^−1^ in control to 34 mg kg^−1^ in T_4_ under normal growth conditions and from 15 mg kg^−1^ in saline control to 25 mg kg^−1^ in T_8_ under saline conditions. Maximum grain Zn was recorded where ZnO-NPs were applied while ZnO in bulk form showed a minimum increase from respective control.

**Figure 5:**
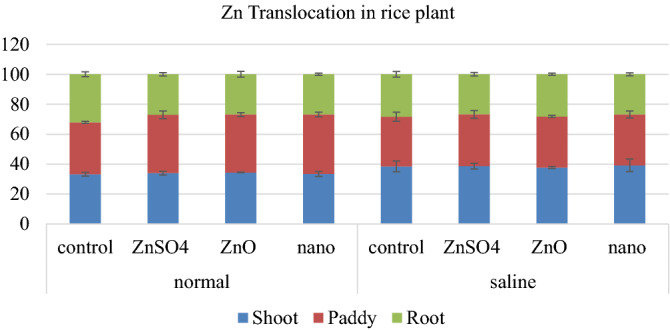
Percentage distribution of Zn translocated in different plant parts of rice at maturity (Each value is a mean, n = 3 statistically significant at p ≤ 0.05, T bars represent ± standard error of means).

Zn uptake per plant also showed the similar trend as Zn concentration. Zn translocation in rice plant in the form of percentage of total plant Zn that is present in each plant part is presented in Fig. 5. Zn translocation from shoot to grain was decreased under saline conditions. But application of Zn significantly improved this translocation from shoot to grain. Although in case of rice there was not a significant difference between different sources in improving Zn translocation from shoot to grain under saline conditions.

## Discussion

Salt stress decreases plant growth and poses physiological depression due to osmotic stress and nutritional imbalance^25^. Zinc is essential for plant growth and grain yield. The Zn is necessary for the fundamental growth processes like cell division and elongation processes^26^. So, adequate Zn supply improved plant height and number of tillers. It also improved chlorophyll concentration in many rice genotypes showing Zn involvement in chlorophyll synthesis^27,28^ and application of Zn positively improved the growth of wheat and rice under the salt stress.

Plant growth parameters have fairly high response with crop salt resilience and Zn supplementation responsiveness under saline conditions. The Zn supply and adequate Zn availability to plant under salt stress improves grain yield of both wheat and rice^29,30^ due to improved water relations, maintenance of higher RWC, turgor and photosynthetic pigments^11,31^. Zinc application in soil through different sources significantly improved plant growth parameters and plant chlorophyll contents. Soil application of ZnSO_4_·7H_2_O improved total chlorophyll content of rice that can be due to Zn involvement in chlorophyll synthesis^27^. Photosynthetic apparatus is one of the main action sites of Zn in plants that can be the reason that chlorophyll contents of plant were improved under Zn application^32,33^. The ZnO-NPs and ZnSO_4_·7H_2_O proved to improve growth characteristics of both wheat and rice under normal and saline conditions. That can be because soil diffusion of ZnSO_4_·7H_2_O is more effective and ZnSO_4_.7H_2_O also cause a bit acidification of the soil zone due to presence of acidic factor SO_4_^2−^^34^. The ZnSO_4_·7H_2_O fertilizer has been reported to promote better Zn diffusion than ZnO based fertilizers^35^ however, in case of ZnO-NPs they have increased colloidal stability and partitioning in soil pore water especially at soil pH of 8 and there is a higher total Zn concentration in soil solution through ZnO-NPs as compared to ZnSO_4_^36^.

Many researchers have reported that plant responds differently to Zn application through different Zn sources, soil Zn status and plant genotype^37^. The ZnO-NPs increased SDW and other growth parameters of cotton plant in saline conditions^38^. Effectiveness of ZnO-NPs over normal size ZnO and ZnSO_4_·7H_2_O has also been reported in chickpea^39^.

High response of ZnO-NPs with respect to other sources can be attributed to lower activity of SOD and peroxidase and thus lower ROS level and lipid peroxidation in ZnO-NPs treated plants^39^. Chemical composition (Na^+^, K^+^ and Zn^2+^) of different plant components of wheat (Table 4) and rice (Table 6) showed a highly significant effect of salt stress on plants. In term of Na^+^ and K^+^ contents rice seems to be more affected by salt stress. Affinity of rice to uptake Na^+^ (2.93 g kg^−1^) was more as compared to wheat (1.42 g kg^−1^); and percentage decrease in rice growth and yield from respective control was also more as compared to wheat due to salt stress. Rice is reported to be less stress tolerant as compared to wheat^25^. However, the response pattern of both crops to Zn application was almost similar in both normal and saline conditions. There was a notable difference in response pattern of different Zn sources. Response of ZnO-NPs was more pronounced in alleviating salt stress and increasing Zn concentration in root, shoot and grain of both crops (wheat and rice) in comparison to other two sources (ZnSO_4_·7H_2_O and ZnO bulk).

In present study, Na^+^ concentration of plant roots, grain and shoot was significantly increased up to two times with respect to respective control in both wheat and rice crops under salt stress conditions. Similarly increase in Na^+^ concentration up to two folds and a decrease in K^+^ concentration and K^+^/Na^+^ ratio under Na_2_SO_4_ salt stress was also reported. That can be due to high Na^+^ uptake by plants resulted in lower K^+^ uptake and secondly cytosolic K^+^ efflux^25,40,41^. High Na^+^ concentration also results in oxidative stress, imbalance in cellular homeostasis, nutrient deficiency, retarded growth and even plant death. Results of current study also showed that with imposition of salinity, Zn concentration in different plant parts was also decreased in both wheat and rice crops which can be due to competition of transport channels to enter into the plant. Under saline conditions, pH of the soil increases due to presence of sodium bicarbonate and availability of Zn decreases^42^. It is also reported that salt stress results in reduced phytosiderophores production and reduced rhizosphere acidification^43^ that results in less nutrient availability to plants. High salt contents in growth medium inhibit the ZnO NP uptake by wheat plant^44^.

There was an increase in K^+^ concentration and K^+^/Na^+^ ratio of shoot root and grain with application of different Zn sources. That can be due to role of Zn in maintaining biomembrane integrity^45^. Preferable binding of Zn to –SH group of membrane protein moiety either direct or close to a –SH group site, reported to protects proteins and phospholipids from disulphide formation and thiol oxidation^46,47^. It was reported that with increasing applied Zn in saline and non-saline conditions, shoot and grain Zn concentration is increased^48,49^. Zinc application helps in maintaining low shoot Na^+^ and hence cytosolic K^+^/Na^+^ ratio is increased. Maintaining a higher K^+^/Na^+^ is a key trait in salt tolerance^50^. Combined effect of salt stress and Zn application showed that salt stress reduces Zn uptake in plants, but progressive application of Zn alleviated the negative impacts of salt stress in wheat^51^. An increased K^+^/Na^+^ ratio is observed through Zn application in wheat and rice respectively under saline conditions^42,43^. The Zn application at any salinity level enhanced Zn concentration in rice shoot. Increase in Zn concentration in plant due to Zn application in soil was the main characteristic that ultimately enhances grain Zn concentration. The Zn fertilizers application has a significant role in enhancing grain Zn concentration of rice^44^.

Zinc concentration in grain can be ranged from 08 to 47 mg kg^−1^ under different Zn application treatments and soil– Zn status^37^. Normally Zn concentration more than 50 mg kg^−1^ in cereal grain is considered enviable to get an optimal beneficial impact on human health to combat malnutrition.

In present study, there was a significant difference among Zn sources towards Zn availability to crop plants. The ZnO-NPs improved Zn contents of shoot and grain at higher rate than other sources. That can be due to difference in chemical reactions that each source goes through in different soil conditions. There exists a difference in diffusion rate of different Zn sources in different soil conditions. The ZnSO_4_·7H_2_O showed more diffusion rate than ZnO due to ionic interactions of Zn^2+^ and SO_4_^2−^^34^. Similar results were reported that zinc sulphate promotes higher Zn diffusion in soil than ZnO based fertilizers^35^. It was also reported that ZnO based fertilizer can be dissolved better at high soil pH and if dispersed well^34^. This can justify the higher response through ZnO-NPs in plant Zn uptake. The ZnO-NPs better responded than ZnSO_4_·7H_2_O at almost 15-time lower dose in peanut growth^52^. Nano-fertilizers or nano-coated fertilizers have increased utilization of delivered nutrients and more site-specific delivery^53^. It is suggested that due to greater dissolution in the rhizosphere, better Zn contents and uptake is induced by ZnO-NPs coated fertilizers as compared to bulk form coating. Also ZnO NPs coatings pose same Ecotoxicological threat as of bulk form^54^. Many researchers reported better performance of ZnO-NPs in different plants such as in maize grains^55^, groundnut^52^, rice^56^ and mung bean^57^.

Nano fertilizers can be a better option in fortifying cereal crop with Zn but in case of soil application there is a need of better understanding of nanoparticles interactions with different soil properties and components. Currently it is critical to develop a thorough understanding of behaviour and fate of ZnO nanoparticles in different soil environments and soil Zn regimes and their possible impact on plant Zn uptake.

## Conclusion

It can be summarized that co-precipitation method for ZnO-NPs preparation can be regarded as a better and somewhat economic option for successful nano synthesis. The ZnO-NPs with size range of 22–60 nm can be synthesized through this method and with better set of conditions size can further be improved.

All used Zn sources effectively alleviated the negative effects of salt stress on plant growth, yield and Zn concentration. Maximum improvement were recorded where ZnO-NPs were applied. It can be concluded that nano-fertilizers when used appropriately with improved set of soil and plant conditions can be a better option in fortifying cereal crop with Zn. A thorough understanding of Zn nanoparticles and soil interactions and their retention and availability need further detailed research under field conditions.

## Data Availability

All obtained data is enclosed with this manuscript.

## References

[1] Hasanuzzaman M, Nahar K, Fujita M, Ahmad P, Azooz M, Prasad M (2013). Plant response to salt stress and role of exogenous protectants to mitigate salt-induced damages. Ecophysiology and Responses of Plants Under Salt Stress.

[2] Tavakkoli E, Rengasamy P, McDonald GK (2010). High concentrations of Na^+^ and Cl^–^ ions in soil solution have simultaneous detrimental effects on growth of faba bean under salinity stress. J. Exp. Bot..

[3] Grattan SR, Grieve CM (1999). Mineral nutrient acquisition and response by plant grown in saline environments. Agric. Ecosyst. Environ..

[4] Rahnama K, Poustini R, Afshari T, Ahmadi A, Alizadeh H (2011). Growth properties and ion distribution in different tissues of bread wheat genotypes (*Triticum aestivum* L.) differing in salt tolerance. J. Agron. Crop Sci..

[5] Khalid N, Hussain M, Aqeel M (2013). Amelioration of adverse effects of stimulated acid rain (SAR) on growth and yield attributes of sunflower (*Helianthus annuus* L.) by growth tonics. Pak. J. Bot..

[6] Rashid A, Ryan J, Alloway BJ (2008). Micronutrient constraints to crop production in the Near East: Potential significance and management strategies. Micronutrient Deficiencies in Global Crop Production.

[7] Cakmak I, Kutman UB (2008). Agronomiv biofortification of cereals with zinc: A review. Eur. J. Soil Sci..

[8] White PJ, Broadley MR (2011). Physiological limits to zinc biofortification of edible crops. Front. Plant Sci..

[9] Brown KH, Wuehler SE, Peerson JM (2001). The importance of zinc in human nutrition and estimation of the global prevalence of zinc deficiency. Food Nutr. Bull..

[10] Ashraf MY, Sarwar G, Ahmad R, Malik KA (2002). Salt tolerance potential in members of Brassicaceae. Physiological studies on water relations and mineral contents. Prospects for Saline Agriculture.

[11] Saeidnejad AH, Kafi M (2013). Alleviative effects of zinc on physiological properties and antioxidants activity of maize plants under salinity stress. Int. J. Agric. Crop Sci..

[12] Jumberi A, Yamada M, Yamada S, Fujiyama H (2001). Salt tolerance of grain crops in relation to ionic balance and ability to absorb microelements. Soil Sci. Plant Nutr..

[13] Nel A, Xia T, Madler L, Li N (2006). Toxic potential of materials at the nano level. Science.

[14] Kalteh M, Alipour ZT, Ashraf S, Aliabadi MM, Nosratabadi AF (2014). Effect of silica nanoparticles on basil (*Ocimum basilicum*) under salinity stress. J. Chem. Health Risks.

[15] FDA (Food and Drug Administration), Washington DC, USA. *Select Committee on GRAS Substances (SCOGS) Opinion: Zinc Salts. CFRSearch.cfm?fr=182.8991* (2015)

[16] Adhikari T, Kundu S, Biswas AK, Tarafdar JC, Rao AS (2015). Characterization of zinc oxide nano particles and their effect on growth of maize (*Zea mays* L.) plant. J. Plant Nutr..

[17] Ahamed AJ, Vijaya Kumar PV (2016). Synthesis and characterization of ZnO nanoparticles by co-precipitation method at room temperature. J. Chem. Pharm. Res..

[18] Athar, T. *Biofortification of Rice in Saline Soils and Salinity Remediation by Application of Nanotechnol*ogy. M.Sc. (Hons.) Thesis, University of Agriculture Faisalabad, Pakistan (2017).

[19] Cullity BD, Stock SR (1956). Elements of X-ray Diffraction.

[20] Naz T, Iqbal MM, Akhtar J, Saqib M (2022). Baseline hydroponic study for biofortification of bread wheat genotypes with iron and zinc under salinity: Growth, ionic, physiological and biochemical adjustments. J. Plant Nutr..

[21] Jones JRJ, Case VW, Westerman RL (1990). Sampling, handling, and analyzing plant tissue samples. Soil Testing and Plant Analysis.

[22] Estefan G, Sommer R, Ryan J (2013). Methods of Soil, Plant, and Water Analysis: A Manual for the West Asia and North Africa Region.

[23] Yang Y, Chen H, Zhao B, Bao X (2004). Size control of ZnO nanoparticles via thermal decomposition of zinc acetate coated on organic additives. J. Cryst. Growth.

[24] Morkoc H, Ozgur U (2009). Zinc Oxide: Fundamentals, Materials and Device Technology.

[25] Munns R, Tester M (2008). Mechanisms of salinity tolerance. Annu. Rev. Plant Biol..

[26] Chang HB, Win LC, Huang HJ (2005). Zinc induced cell death in rice (*Oryza sativa* L.) roots. Plant Growth Regul..

[27] Chen W, Yang X, He Z, Feng Y, Hu F (2008). Differential changes in photosynthetic capacity, 77 K chlorophyll fluorescence and chloroplast ultrastructure between Zn efficient and Zn-inefficient rice genotypes (*Oryza sativa* L.) under low zinc stress. Physiol. Plant..

[28] Weisany W, Sohrabi Y, Heidari G, Siosemardeh A, Ghassemi-Golezani K (2011). Physiological responses of soybean (*Glycine max* L.) to zinc application under salinity stress. Aust. J. Crop Sci..

[29] Rani S, Sharma MK, Kumar N, Neelam (2019). Impact of salinity and zinc application on growth, physiological and yield traits in wheat. Curr. Sci..

[30] Daneshbakhsh B, Khoshgoftarmanesh AH, Shariatmadari H, Cakmak I (2013). Phytosiderophore release by wheat genotypes differing in zinc deficiency tolerance grown with Zn-free nutrient solution as affected by salinity. J. Plant Physiol..

[31] Jabeen N, Ahmad R (2012). Improvement in growth and leaf water relation parameters of sunflower and safflower plants with foliar application of nutrient solutions under salt stress. Pak. J. Bot..

[32] Bonnet M, Camares O, Veisseire P (2000). Effects of zinc and influence of *Acremonium lolii* on growth parameters, chlorophyll fluorescence and antioxidant enzyme activities of ryegrass (*Lolium perenne* L. cv Apollo). J. Exp. Bot..

[33] Cherif J (2010). Analysis of in vivo chlorophyll fluorescence spectra to monitor physiological state of tomato plants growing under zinc stress. J. Photochem. Photobiol..

[34] Santos WO (2019). Zinc diffusion and availability affected by different sources in soils of contrasting textures. J. Agric. Sci..

[35] Mattiello EM (2017). Sulfur and zinc availability from co-granulated Zn-enriched elemental sulfur fertilizers. J. Agric. Food Chem..

[36] Baddar ZE, Matochaa CJ, Unrine JM (2019). Surface coating effects on the sorption and dissolution of ZnO nanoparticles in soil. Environ. Sci. Nano.

[37] Wissuwa M, Ismail AM, Graham RD (2008). Rice grain zinc concentrations as affected by genotype, native soil-zinc availability, and zinc fertilization. Plant Soil..

[38] Hussein MM, Abou-Baker NH (2018). The contribution of nano-zinc to alleviate salinity stress on cotton plants. R. Soc. Open Sci..

[39] Burman U, Saini M, Kumar P (2013). Effect of zinc oxide nanoparticles on growth and antioxidant system of chickpea seedlings. Toxicol. Environ. Chem..

[40] Tester M, Davenport R (2003). Na^+^ tolerance and Na^+^ transport in higher plants. Ann. Bot..

[41] Cabot C, Sibole JV, Barceló J, Poschenrieder C (2014). Lessons from crop plants struggling with salinity. Plant Sci..

[42] Gupta RK, Singh RR, Tanji KK (1990). Phosphorus release in sodium ion dominated soils. Soil Sci. Soc. Am. J..

[43] Yousfi S, Wissal M, Mahmoudi H, Abdelly C, Gharsalli M (2007). Effect of salt on physiological responses of barley to iron deficiency. Plant Physiol. Biochem..

[44] Stewart J (2015). Salts affect the interaction of ZnO or CuO nanoparticles with wheat. Environ. Toxicol. Chem..

[45] Marschner H (1995). Mineral Nutrition of Higher Plants.

[46] Chvapil M (1973). New aspects in the biological role of zinc: A stabilizer of macromolecules and biological membranes. Life Sci..

[47] Sharma PN, Kumar N, Bisht SS (1994). Effect of zinc deficiency on chlorophyll content, photosynthesis and water relations of cauliflower plants. Photosynthetica.

[48] Fathi A, Zahedi M, Torabian S (2017). Effect of interaction between salinity and nanoparticles (Fe_2_O_3_ and ZnO) on physiological parameters of *Zea mays* L.. J. Plant Nutr..

[49] Moradi S, Jahanban L (2018). Salinity stress alleviation by Zn as soil and foliar applications in two rice cultivars. Commun. Soil Sci. Plant Anal..

[50] Shabala S, Pottosin I (2014). Regulation of potassium transport in plants under hostile conditions: Implications for abiotic and biotic stress tolerance. Physiol. Plant..

[51] Rajput VD (2021). Coping with the challenges of abiotic stress in plants: New dimensions in the field application of nanoparticles. Plants.

[52] Prasad TNVKV (2012). Effect of nanoscale zinc oxide particles on the germination, growth and yield of peanut. J. Plant Nutr..

[53] Chugh G, Siddique KHM, Solaiman ZM (2021). Nanobiotechnology for agriculture: Smart technology for combating nutrient deficiencies with nanotoxicity challenges. Sustainability.

[54] Milani N (2015). Fate of zinc oxide nanoparticles coated onto macronutrient fertilizers in alkaline calcareous soil. PLoS ONE.

[55] Pandey AC, Sanjay SS, Yadav RS (2010). Application of ZnO nanoparticles in influencing the growth rate of (*Cicer arietinum*). J. Exp. Nanosci..

[56] Boonyanitipong P, Kositsup B, Kumar P, Baruah B, Dutta J (2011). Toxicity of ZnO and TiO_2_ nanoparticles on germinating rice seed (*Oryza sativa* L.). Int. J. Biosci. Biochem. Bioinform..

[57] Jayarambabu M, Sivakumari B, Rao KV, Prabhu YT (2014). Germination and growth characteristics of mungbean seeds affected by synthesized ZnO nanoparticles. Int. J. Curr. Eng. Technol..

